# Online interprofessional education materials through a community learning program during the COVID 19 pandemic in Chile

**DOI:** 10.3352/jeehp.2022.19.6

**Published:** 2022-03-24

**Authors:** Sandra Oyarzo Torres, Mónica Espinoza Barrios

**Affiliations:** Department of Education in Health Sciences and Undergraduate Management, Faculty of Medicine, University of Chile, Santiago, Chile; Hallym University, Korea

**Keywords:** Chile, COVID-19, Health education, Health occupations students, Interprofessional relations

## Abstract

This article aims to share the online collaborative experience of interprofessional teamwork among healthcare undergraduate students based on community learning during the coronavirus disease 2019 (COVID-19) pandemic in Chile. This experience took place in 48 different communities in Chile from November 10, 2020 to January 12, 2021. It was a way of responding to the health education needs of the community when the entire Chilean population was in confinement. Students managed to adapt to the COVID-19 pandemic despite the challenges, including internet connectivity problems and the limited time available to do the work. The educational programs and videos shared in this article will be helpful for other interprofessional health educators to implement the same kind of program.

## Introduction

### Background

Interprofessional courses, in particular, represent an inter-curricular effort that has benefits for students and academics involved in the health professions, and especially for people, families, and communities that are at the center of health care. There was an interprofessional program to support community residents who had no prompt access to healthcare teams by responding to sanitary emergencies caused by the coronavirus disease 2019 (COVID 19) pandemic. This program was done through online educational interventions in healthcare seeking to satisfy community needs. It also pursued the strengthening of students’ skills for social commitment, respect for diversity, and a rights approach. It was conducted in collaboration with community leaders. Information about the interprofessional education program can be accessed through the Faculty of Medicine of the University of Chile available at: http://www.medicina.uchile.cl/; http://formacioncomun.med.uchile.cl. This study was conducted among 650 students from the 8 healthcare undergraduate programs (medicine, nursing, midwifery, speech therapy, physical therapy, occupational therapy, medical technology, nutrition, and dietetics), grouped into 28 teams with 28 professors from the Faculty of Medicine of the University of Chile, and 48 leaders from social organizations. This experience took place in 48 different communities of Santiago, and the rest of Chile during the COVID 19 pandemic, from November 10, 2020 to January 12, 2021 (Supplement 1).

### Objectives

This article aimed to share the collaborative experience of interprofessional teamwork, including programs and video files of education for community residents.

## Teaching and learning activities

In 2020, due to the COVID 19 pandemic, all activities were performed online (Table 1). This was done to protect the health of students, professors, and community residents. The experience made it possible to integrate teaching and community engagement. Throughout these experiences, students developed teamwork skills, respect for the professional role of each member of the healthcare team and other professions and disciplines, and integration with the local community laborers, in collaboration with social leaders and teachers. The methodology used was to carry out a diagnosis of educational needs detected in the community through online focus groups, problem trees, brainstorming, and online questionnaires. After the information was collected, a Gantt chart was made with the development of the online health education project, in conjunction with community leaders based on the educational needs detected in the population among which the intervention would be carried out.

## Challenges in implementing the online interprofessional education experience based on community learning

Students had to carry out an online healthcare educational project based on community needs related to the COVID-19 pandemic care such as hand washing and alcohol gel disinfection, mental health care during the pandemic, and responsible pet ownership. During this experience, effective communication stood out, in the virtual framework with people and groups in different areas of this intervention, as participants adhered to ethical and bioethical principles in their praxis. At the same time, the experience set out new challenges related to accessibility and coordination with community leaders via new communication technologies such as Zoom, Meets, WhatsApp, Instagram, YouTube, and infographics.

## Implementing the online interprofessional education experience based on community learning

Students inquired into their former knowledge associated with disciplinary and experiential spheres. They also faced situational contexts, which presented an opportunity to apply that knowledge to community education during the COVID-19 pandemic. The experience enabled dynamism and the acquisition of teamwork skills through a virtual modality. Students also valued the contribution of different professionals and community roles to face a problem that had characteristics transferable to their future professional performance. Although the experience was positive, it would still be premature to draw a definitive conclusion regarding the impact of this modality of work on the skills acquired through interprofessional education [1].

The material developed by students was shared with the community for educational purposes: educational videos, audio reports, and infographics were generated and published on Instagram (Supplements 2–4).

## Conclusion

Students were able to adapt to the COVID 19 pandemic situation to educate community residents despite challenges, including a lack of internet connectivity and limited time available to do the work. The flexibility of all team members and their commitment to carry out the community project stood out as important factors. The implementation of this program was achieved by planning, articulating, implementing, and assessing an intervention that became useful for the community and the team. The educational materials and videos shared in this article will be helpful for other interprofessional health educators to implement the same kind of program.

## Figures and Tables

**Table 1.** Activity schedule of the 8-week online community program

Weeks	Activities	Details
1st week	Introduction to the course	Introducing each student and teacher, asking them how they and their families are doing, what difficulties they are having, what they expect from this course. The teacher talks to students about the course syllabus and evaluation system.
2nd week	Building the team together with community leaders	Introducing each student, teacher, and community leader to get to know each other to plan the community intervention
3rd week	Determining the health-related educational needs within an objective community group; completing a bibliographical review	Participating with their team in the detection of community health educational needs.
4th week	Planning the healthcare educational intervention in reply to the diagnoses made in the community.	Defining, with their work teams: purpose, general and specific objectives, and the topics to be treated during the intervention, in line with what had been planned.
5th week	Building methodologies to be used during the healthcare educational intervention	Building, in collaboration with their team, teacher, and community leader methodologies to be used during the healthcare educational intervention, supporting such decisions, defining the necessary resources to the development of the healthcare educational intervention, and the type of evaluation to be employed according to the previously set objectives. Methodologies for community work: small group work; online interviews; problem tree; brainstorming; infographics; videos; and communication technologies such as Zoom, Meets, WhatsApp, Instagram, YouTube.
6th week	Performing the first session	Students, teachers, and community leaders used methodologies appropriate to the objectives and contexts, contributing with their professional knowledge to their performance of the first and second session.
7th week	Performing the second session	Students, teachers, and community leaders used methodologies appropriate to the objectives and contexts, contributing with their professional knowledge to their performance of the first and second session.
8th week	Evaluating the different dimensions of the interprofessional healthcare educational intervention	Students, teachers, and community leaders participated in the evaluation of the intervention.

## Supplementary materials

Supplementary files are available from Harvard Dataverse: https://doi.org/10.7910/DVN/GOVLBA

Supplement 1. Interprofessional education course program at the Faculty of Medicine, University of Chile, 2021 (in Spanish).

Supplement 2. Example of Interprofessional education infographics “Infografía Tenencia Responsable de mascotas” used by students to educate community groups (in Spanish)The infographics developed by the students included different educational topics requested by the community, such as responsible pet ownership

Supplement 3. Example of Interprofessional educational video Disinfection with Alcohol Gel used by students to educate community groups (in Spanish). The videos developed by the students allowed them to teach about different topics such as COVID-19 and the use of vaccines.

Supplement 4. Example of an interprofessional educational video on mental health used by students to educate community groups (in Spanish). The mental health videos were very important to the community, as the confinement due to the COVID-19 pandemic generated the need for education by students in this area.

Supplement 5. Audio recording of the abstract.
