# Supplementary material for: Online interprofessional education materials through a community learning program during the COVID 19 pandemic in Chile
Source: J Educ Eval Health Prof. 2022 Mar 24;19:6. doi: 10.3352/jeehp.2022.19.6 (PMC9086809; doi:10.3352/jeehp.2022.19.6)
Supplement: Supplementary file 1 — Supplement 1. Interprofessional education course program at the Faculty of Medicine, University of Chile, 2021 (in Spanish). [file jeehp-19-06-suppl1.docx]

**P R O G R A M M I I M 2 ONLINE COURSE 2020**

| **Academic Unit:** Common Formation/Direction of Undergraduate Studies  **Course Name :** Multiprofessional Interdisciplinary Integrated Module 2  **Code:** MIO 4020505002-2  **Career:** Nursing, Speech Therapy, Kinesiology, Medicine, Nutrition, Obstetrics, Medical Technology, Occupational Therapy.  **Type of course:** Compulsory  **Area of study:** Common Formation  **Level:** Fourth and fifth year  **Semester:** Eighth  **Year: 2020**  **Schedule:** Tuesdays from 15:00 - 18:00 pm  **Requirements:** MIIM I  **Number of credits:** 5 credits (135 hours)  **Synchronous work hours: 27 hrs**  **Asynchronous working hours :** 27 hrs  **Number of students:** 650 |
| --- |

| **TEAM RESPONSIBLE FOR THE MIIM 2 PROGRAM:**   - Common Formation Coordinator: Prof. Mónica Espinoza Barrios. - MIIM General Coordinator: Prof. Sandra Oyarzo Torres - Community Extension Director. Faculty of Medicine: Prof. Hernán Aguilera - Department of Primary Care and Family Health: Prof. Claudia Muñoz - Cerro Navia Health Department: Prof. Alvaro Besoain - Health Social Leaders Coordinator: Mr. Juan Sancho. President of the Health Council. North Area - President of the Council of Civil Society Organizations (COSOC) of the SSMN. National Councilor COSOC of MINSAL. Coordinator of ECOSS de Barrio. RM and national leader of the Citizen Participation Council. MINSAL. - Pudahuel Leaders Coordinator: Mr. Jorge Barrientos. Leader of Environmental Cultural Centers and JVV. Member of consultative councils at the level of the consulting room. Servicio Occidente in Santiago and Metropolitano Nacional. Member of the ethics committee of Servicio Salud Occidente. Metropolitan Region. Member of community radio and television networks. Health and environmental leader in the province of Petorca.       MIIM Teaching Secretary: Camila Navarro miim@med.uchile.cl  Telephone contact: 229786502 |
| --- |

| N° | **University Teacher/Facilitator** | **Academic Unit** | **Hours** |
| --- | --- | --- | --- |
| 1 | Prof. Bernardo Jil Vargas. | Nursing School | 60 |
| 2 | Prof. Ximena Rivera | Nursing School | 60 |
| 3 | Prof. Felipe Velásquez | ICBM | 60 |
| 4 | Prof. Sussy Tapia Álvarez. | Women's and Newborn Health Promotion Dept. | 60 |
| 5 | Prof. Paola Gaete Hermosilla. | Women's and Newborn Health Promotion Dept. | 60 |
| 6 | Prof. Patricia Gálvez Espinoza. | Nutrition Dept. | 60 |
| 7 | Prof. Tomás Hernández González. | Kinesiology School | 60 |
| 8 | Prof. Américo López Rivera. | ICBM | 60 |
| 9 | Prof. Mónica Espinoza Barrios. | DECSA/ Direction of Undergraduate Studies | 60 |
| 10 | Prof. Sandra Oyarzo Torres. | DECSA/ Direction of Undergraduate Studies | 60 |
| 11 | Prof. Paula Soto | Occupational Therapy and Occupational Science Dept. | 60 |
| 12 | Prof. Luis Romero | Department of Speech Therapy | 60 |
| 13 | Prof. Marcelo Vásquez León. | Dept. Primary Care and Family Health | 60 |
| 14 | Prof. Claudia Muñoz Jofré. | Dept. Primary Care and Family Health |  |
| 15 | Prof. María José Jorquera González. | Dept. Primary Care and Family Health | 60 |
| 16 | Prof. Johnny Acevedo Ayala. | Dept. Primary Care and Family Health | 60 |
| 17 | Prof. Eliana Espinoza Alarcón. | Dept. Primary Care and Family Health | 60 |
| 18 | Prof. Javier Chacón Rivas. | Dept. Primary Care and Family Health | 60 |
| 19 | Prof. Hernán Aguilera Martínez. | Community Extension Director. Faculty of Medicine | 60 |
| 20 | Prof. Álvaro Besoaín Saldaña. | Health Dept. Health Dept. CESFAM Cerro Navia | 60 |
| 21 | Prof. Matías Goyenechea Hidalgo. | Medical Technology Dept. | 60 |
| 22 | Prof. Germán Ebensperger Darrouy. | ICBM | 60 |
| 23 | Prof. Paulina Larrondo Valderrama | DIGEN Health Coordinator | 60 |
| 24 | Prof. Marcelo Saldías | Department of Speech Therapy | 60 |
| 25 | Prof. Alejandro Candia Henríquez. | Women's and Newborn Health Promotion Dept. | 60 |
| 26 | Prof. Cristóbal Catalán | Occupational Therapy and Occupational Science Dept. | 60 |
| 27 | Prof. Ana Cubillos Galdámez | Dept. Medical Technology | 60 |
| 28 | Prof. Tania Espinoza | Nursing Dept. | 60 |

1. **Historical Context of the MIIM Program:**

The development of the Curricular Innovation Process carried out in the Faculty of Medicine since 2007, implied a stage of survey of training needs in the eight careers of Health, which allowed evidencing the need to establish common curricular spaces that would allow the development of generic competencies transversal to the eight careers taught in the Faculty, and would enhance the integration of knowledge, both at interdisciplinary and multiprofessional level. In this context, it was agreed to build a Common Training Program, with a total of 36 credits, to which all health careers will be progressively incorporated as they implement their competency-based curriculum. This program included 15 credits of General Training, 12 credits of English language training and 9 credits for two Multiprofessional Interdisciplinary Integration Modules (MIIM). Both modules will enhance teamwork, the first one with an emphasis on the integration of basic science knowledge and the second one on Public Health. They will be taught in the fourth and eighth semester respectively. Currently MIIM I is located in the fifth semester and MIIM II in the eighth semester of 7 careers and Medicine in the tenth semester.

2.  **Construction process of MIIM II**:

MIIM is a teaching innovation assumed by the Faculty in order to provide its students with the dual opportunity to learn to work together with students from other professions in the health field, and to mobilize the knowledge already acquired during their time at the University.

The curricular assumptions are affirmed in the idea of curricular integration, since the curriculum is an instrument for the construction of a professional identity. Being competent implies selecting, mobilizing and articulating resources of diverse origin and nature to produce solutions to problems that are specific to the profession. Integration is a key feature of professional practice and should therefore be considered as such in the training proposal.

In pedagogical terms, early opportunities for the integration of knowledge will allow students to find the meaning and significance of the learning, particularly the initial ones that are not so clearly linked to the profession. At the same time, in the MIIM model, integration appears not only in its interdisciplinary perspective but also in the knowledge-professional practice relationship and between the different professions being studied by the participants.

3. **Rationale for the MIIM course on Interprofessional Education in Health Care**

In 2010, the World Health Organization (WHO), in the framework of action on interprofessional education and collaborative care, highlighted the importance of interprofessional education to promote sustainable models based on interdisciplinary teamwork for the care of people in health.

Interprofessional education (IPE) aims to improve collaboration between different types of health and social care professionals to facilitate collaboration and care. As defined by CAIPE (Centre for the Advancement of Interprofessional Education https://www.caipe.org) it is achieved when: "students from two or more professions learn with each other, from each other and about each other, to collaborate in professional practice."

Interprofessional Education has been widely introduced as an important topic to be addressed from different perspectives in the training of health professionals, with the aim of providing comprehensive and people-centered care, improving the quality of life of communities. The incorporation of interprofessional curricular models in the training plans of health students has been promoted as an important advance to respond to the current demands of health systems and the community.

The University of Chile, in its permanent commitment to the WHO guidelines, responded to the need to implement collaborative learning among the eight health careers in the Faculty of Medicine by creating the Multiprofessional Interdisciplinary Integrated Modules (MIIM 1 and MIIM 2). These courses are mandatory for all health students during their third and fourth year (7 careers) fifth year (Medicine career) year of discipline-specific studies. During these courses students from Obstetrics, Nursing, Occupational Therapy, Kinesiology, Medical Technology, Speech Therapy, Medicine and Nutrition must work together to achieve the course objectives based on teamwork. The MIIM 1 and MIIM 2 modules in particular, represent an intercurricular effort that benefits the students and faculty of the University of Chile involved in the health professions.

| **SOCIAL LEADER** | **ORGANIZATION** | **COMMUNITY** | **UNIVERSITY TEACHER FACILITATOR** | **TEAMS** |
| --- | --- | --- | --- | --- |
| Nicole Castillo  Verónica Belmar | Confirmado, Huaquén | Petorca | Prof. Bernardo Gil Vargas. | **Sección 1**  **Grupo 1 y Grupo 2** |
| Manuel Berrios | Confirmado | Papudo | Prof. Tania Espinoza | **Sección 2**  **Grupo 3** |
| Víctor | Confirmado, Teniente Merino | Pudahuel | Prof. Tania Espinoza | **Sección 2**  **Grupo 4** |
| Gladys | Confirmado, El Comendador | Pudahuel | Prof. Paola Gaete Hermosilla | **Sección 3**  **Grupo 5** |
| Gladys | Confirmado, Victoria | Pudahuel | Prof. Paola Gaete Hermosilla | **Sección 3**  **Grupo 6** |
| Nina Araya | Confirmado, Monseñor Larraín | Pudahuel | Prof. Mónica Espinoza Barrios | **Sección 4**  **Grupo 7** |
| Bárbara Astudillo Artificio | Confirmado, Cabildo y Petorca | Petorca | Prof. Mónica Espinoza Barrios | **Sección 4**  **Grupo 8** |
| Jano Marín | Confirmado, Jardín Lo Espejo | Pudahuel | Prof. Sandra Oyarzo Torres | **Sección 5**  **Grupo 9** |
| David Torres | Confirmado, Santa Beatriz | Pudahuel | Prof. Sandra Oyarzo Torres | **Sección 5**  **Grupo 10** |
| Melisa Elgueta Gómez | Confirmado, Los Lagos | Petorca | Prof. Alejandro Candia Henríquez | **Sección 6**  **Grupo 11** |
| Francisco González | Confirmado, Santa Corina | Pudahuel | Prof. Alejandro Candia Henríquez | **Sección 6**  **Grupo 12** |
| Margarita Sandoval Carrasco | Confirmado | Papudo | Prof. Hernán Aguilera | **Sección 7**  **Grupo 13** |
| Daniela Rivera  María Paz Ariztía | Confirmado | Cerro Navia | Prof. Alvaro Besoain | **Sección 8**  **Grupo 14** |
| Alexandra Rivera  Marcelo Bustos | Confirmado | Cerro Navia | Prof. Alvaro Besoain | **Sección 8**  **Grupo 15** |
| Jorge Barrientos | Confirmado | Cerros del Romeral | Prof. Américo López | **Sección 9**  **Grupo 16** |
| Jorge Barrientos | Confirmado | Cerros del Romeral | Prof. Américo López | **Sección 9**  **Grupo 17** |
| Con Dirigente | Confirmado | Depto APS y Salud Familiar. U de Chile | Prof. Claudia Muñoz  Prof. María Jorquera | **Sección 10**  **Grupo 18** |
| Mitalia Sepúlveda | Confirmado | Conchalí | Prof. Patricia Gálvez | **Sección 11**  **Grupo 19** |
| Claudia | Confirmado | El Rayado, Petorca | Prof. Patricia Gálvez | **Sección 11**  **Grupo 20** |
| Con Dirigente | Confirmado | Depto APS y Salud Familiar. U de Chile | Prof. Javier Chacón | **Sección 12**  **Grupo 21** |
| Lorna | Confirmado | Los Molles  Villa Huaquén, Petorca | Prof. Jhonny Acevedo Ayala | **Sección 13**  **Grupo 22** |
| Valeria Altamirano | Confirmada | La Higuera, El Carmen y Cabildo. Petorca | Prof. Matías Goyenechea | **Sección 14**  **Grupo 23** |
| Pablo | Confirmado | El Mirador, Petorca | Prof. Matías Goyenechea | **Sección 14**  **Grupo 24** |
| Marcos Cruz Olavarría | Confirmado | Conchalí | Prof. Germán Ebensperger | **Sección 15**  **Grupo 25** |
| Catalina Aliste | Confirmado | Conchalí | Prof. Germán Ebensperger | **Sección 15**  **Grupo 26** |
| Jacqueline Torres | Confirmada | El Mirador de la Ligua | Prof. Marcelo Saldías | **Sección 16**  **Grupo 27** |
| Víctor Hugo Pérez | Confirmado | Conchalí | Prof. Marcelo Saldías | **Sección 16**  **Grupo 28** |
| Manuel Berríos | Confirmado | Papudo | Prof. Ximena Rivera | **Sección 17**  **Grupo 29** |
| Margarita Sandoval | Confirmado | Papudo | Prof. Ana Cubillos | **Sección 18**  **Grupo 30** |
| Álvaro Núñez | Confirmado | Pullally, Petorca | Prof. Eliana Espinoza  Prof. Marcelo Vásquez | **Sección 19**  **Grupo 31** |
| Omar Fuentes | Confirmado | Conchalí | Prof. Sussy Tapia | **Sección 20**  **Grupo 32**  |
| Marcela Escobar | Confirmado | Pullally, Petorca | Prof. Sussy Tapia | **Sección 20**  **Grupo 33** |
| Muriel Duarte | Confirmado | Conchalí | Prof. Tomás Hernández | **Sección 21**  **Grupo 34** |
| Cristián Toloza | Confirmado | Conchalí | Prof. Tomás Hernández | **Sección 21**  **Grupo 35** |
| Enrique Arrieta | Confirmado | Conchalí | Prof. Paula Soto | **Sección 22**  **Grupo 36** |
| Roberto González | Confirmado | Conchalí | Prof. Paula Soto | **Sección 22**  **Grupo 37** |
| Ingrid Miranda | Confirmada | Pichicuy | Prof. Luis Romero | **Sección 23**  **Grupo 38** |
| Jessica | Confirmado | Conchalí | Prof. Luis Romero | **Sección 23**  **Grupo 39** |
| Jacqueline Clark | Confirmado | Lampa | Prof. Cristóbal Catalán | **Sección 24**  **Grupo 40** |
| Julio Ramírez | Confirmado | Independencia | Prof. Cristóbal Catalán | **Sección 24**  **Grupo 41** |
| Mario Aedo | Confirmado | Recoleta | Prof. Felipe Velásquez | **Sección 25**  **Grupo 42** |
| Eduardo Juri Nahas | Confirmado | Recoleta | Prof. Felipe Velásquez | **Sección 25**  **Grupo 43** |
| Pía Villa | Confirmada | Huaquén, Los Molles | Prof. Paulina Larrondo | **Sección 26**  **Grupo 44** |
| Susana Hernández | Confirmado | La Florida | Prof. Paulina Larrondo | **Sección 26**  **Grupo 45** |
| Mónica Ramirez  Aylin Sepúlveda | Confirmado | Cerro Navia | Prof. Alvaro Besoain | **Sección 27**  **Grupo 46** |
| Camila Maturana  Ximena Yañez | Confirmado | Cerro Navia | Prof. Alvaro Besoain | **Sección 27**  **Grupo 47** |
| Fundación Galia Díaz Riffo  ANFUCULTURA  Jorge González | Confirmado | CCRA Huertos FAMILIARES  TIL - TIL | Prof. Héctor Díaz Prof.  Sandra Oyarzo Torres | **Sección 28**  **Grupo 48** |

| **FORMATIVE PURPOSE:**  This course aims to develop in students skills for cooperative and interprofessional work, through educational interventions in health tending to meet the needs of the community, enhancing the development of competencies linked to: social commitment, inclusion, and respect for diversity, gender approach and rights, in the work together with community Leaders.  It is the second instance of work in interprofessional education, which allows students to recognize themselves as part of a health team, in a respectful work environment that promotes people-centered health care.  During this year 2020, due to the Covid 19 pandemic, all activities will be developed in online work mode, in order to safeguard the health of students, teachers and community.  It is curricular related to MIIM I of the fifth semester. |
| --- |
| **COMPETENCES OF THE SCHOOL OF MEDICAL TECHNOLOGY TO WHICH MIIM II CONTRIBUTES**  Comp.3.Use tools to approach people according to their individual characteristics, their group and social context to interact in a relevant way to the situation and to obtain the necessary information to decide the actions to develop in their professional field.  SC 3.1 Effectively using verbal, body and written communication.  **COMPETENCIES SCHOOL OF NUTRITION TO WHICH MIIM II CONTRIBUTES**  Comp.3 Addressing food-nutritional problems of individuals, collectives and communities, within the framework of public health policies and other social protection policies, with a humanistic and interdisciplinary approach, considering local availability, access to food and social determinants of health, facilitating the active participation of the community, in a given territory respecting ethical and bioethical principles.  SC. 11 Developing skills and attitudes that make it possible to effectively address the problems of food, nutrition and Health, with a collective and community approach, with emphasis on the development of the territories.  Comp .5 Execute, monitor and evaluate food and nutrition education and communication programs, oriented to contribute to a better health of the population, taking into account the age, socioeconomic and cultural diversity of the target audiences and respecting ethical and bioethical principles.  SC. 5.2 Implementing in team work, a community intervention project in Food, Nutrition and Health previously planned, respecting ethical principles.  SC. 5.3 Evaluate in team work, a previously planned community intervention project in Food, Nutrition and Health, respecting ethical principles.  **COMPETENCIES SCHOOL OF MIDWIFERY TO WHICH MIIM II CONTRIBUTES**  Comp 4 Work in a team, identifying potentialities and delimiting own responsibilities as well as those of the rest of the group, prioritizing the interests of the group before their own, for the achievement of a common task, in the terms, deadlines and conditions set in the different contexts of their training.  SC 4.1 Incorporating elements of personal development that allow him/her to integrate into teamwork.  SC 4.2 Recognizing personal characteristics that allow him/her to develop a transversal leadership in work teams.  SC 4.3 Enhancing personal characteristics that allow him/her to become a positive leader in health teams.  Comp.3 Develop actions that evidence citizen commitment with the aim of favoring the construction of a better society, understood as one in which individual and social group responsibility plays a leading role, for the detection of needs and the elaboration of proposals and interventions that contribute to the common good.  SC 3. Identifying the impact of the health team's actions in the community.  **COMPETENCIES SCHOOL OF OCCUPATIONAL THERAPY TO WHICH MIIM II CONTRIBUTES**  Comp.6 Establish assertive, empathetic, and honest professional and interpersonal relationships with individuals and groups, creatively and with leadership in different contexts and environments.  SC 6.1 Understanding the various elements involved in professional work and interpersonal relationships in therapeutic intervention with individuals and groups.  SC 6.4 Actively collaborating and prioritizing the interests of the collective before their own, for the achievement of a common task.  **COMPETENCES SCHOOL OF KINESIOLOGY TO WHICH MIIM II CONTRIBUTES.**  3rd Competence.  3.2. Performs proactively and effectively in a team.  **COMPETENCES SCHOOL OF PHONOAUDIOLOGY TO WHICH MIIM II CONTRIBUTES**  C2: Integrates work teams in order to develop actions that contribute to the understanding and solution of phonoaudiological situations.  SC2.1: Acting with commitment in work teams.  SC2.2: Strengthening the understanding and building agreements between the different actors.  **SCHOOL OF NURSING COMPETENCIES TO WHICH MIIM II CONTRIBUTES**  SC.1.1.15: Actively integrating (with different actors of the health team) in multiprofessional health teams and multisectorIal teams in the solution of individual, family and community health needs.  **EDUCATION DOMAIN**  C.4.2 Participate in the development of multiprofessional and multidisciplinary educational programs to build answers to health needs, from an integral approach in the different areas of performance.  SC 4.2.2 Joining work teams from professional knowledge in the development of community health projects.  **COMPETENCIES MEDICAL SCHOOL TO WHICH MIIM II PAYS TRIBUTE**  2. SOCIAL-ETHICAL DOMAIN  C1. Communicates effectively with people of all ages and conditions, whether they are patients, members of the work team or the community, highlighting their active listening skills, assertiveness, empathy and willingness to help, to improve or preserve health conditions.  .  ● Sub competence 1.3 Establishes an empathetic, cordial and trusting relationship with the patient, his/her family, the health team and the general population.  ● Sub competence 1.4 Participates in teaching the patient, family, community and health team, sharing and putting into practice their knowledge and skills.  C2. Integrates work teams exercising participatory and collaborative leadership, with flexibility and adaptability to change, with a permanent attitude of service and commitment in the various areas in which he/she works.  ● Sub competence 2.1 Contributes to the growth and overcoming of weaknesses, and enhances personal strengths, as well as those of the health team and the community, which directly or indirectly influence the health situation.  ● Sub competence 2.2 Actively contributes with flexibility and adaptability to resolve situations of conflict and uncertainty generated in the health team, seeking the solution in a participatory and collaborative manner.  C6. Participates in the formulation and promotion of solutions in the face of diverse community needs in all areas in which he/she works, which directly or indirectly affect the health status of socially vulnerable groups, seeking the general welfare.  ● Sub competence 6.1 Proposes interventions based on assessments of the diverse health needs of the community in a state of social vulnerability.  **4. PUBLIC HEALTH DOMAIN**  C2 Performs diagnosis of population health situation at the local level, taking into consideration existing local, regional and national information or generating it if necessary.  ● Sub competence 2.1 Uses sources of information related to the population health level, considering their advantages and limitations, to describe a health situation.  ● Sub competence 2.2 Gathers secondary information on health determinants, to describe the health situation at the local level.  ● Sub competence 2.3 Constructs and interprets health level indicators.  ● Sub competence 2.4 Applies the epidemiological description model to the study of health problems in his/her community, using the variables of place, time and person.  ● Sub competence 2.7 Systematizes the relevant information, expressing it in a health situation diagnosis.  **5. GENERIC CROSS-CUTTING DOMAIN**  Sub competence 2.1 Evaluates in a critical and reflective manner his/her work and that of others in different areas of performance, based on theoretical, methodological and ideological criteria.  Sub competence 2.2 Generates strategies aimed at implementing improvement or problem-solving actions in different areas of professional performance. |
| **LEARNING OUTCOME OF THE COURSE:**  1. Carries out educational project in health in an online way, in conjunction with community leaders from the educational needs detected in the population to intervene, forming part of an interprofessional team of students working cooperatively with the assigned community.  2. Present information about the work done through digital media that are available. |

| **WORK PLAN** | | |
| --- | --- | --- |
| **HEALTH EDUCATION INTERVENTION** | 1. Determine health education needs in a community target group. | 1.1 Participates with his/her work team in the detection of educational needs of the assigned community, to be considered in the health education intervention. |
|  | 2. Conduct a literature or evidence review to support the educational intervention in health. | 2.1 Participates with his/her work team in the search for information and the elaboration of the theoretical framework of the educational topics to be dealt with. |
|  | 3. Plan the health education intervention to be carried out in response to the diagnosis made. | 3.1 Defines with his/her work team: the purpose, the general and specific objectives and the topics of the educational intervention in health, coherent with the educational intervention carried out.  3.2 Constructs with his/her work team the methodologies to be used in the development of the educational intervention in health, basing such decisions.  3.3 Defines the necessary resources for the development of the educational intervention in health.  3.4 Establishes with his/her work team the type of evaluation to be used according to the proposed objectives. |
|  | 4. Execute the health needs educational intervention | 4.1 Conducts sessions together with his/her work team, with methodologies relevant to the target group and its context characteristics, contributing from his/her professional knowledge.  4.2 Installs effective communication in the framework of virtual work with individuals and groups in the different areas of intervention, respecting ethical and bioethical principles in their actions. |
|  | 5. Evaluate the health education intervention carried out | 5.1 Performs evaluation of the different dimensions of the educational intervention in health, considering the participation of all actors in this process. |
|  | 6. Support their educational intervention in health in a coherent manner with the diagnosis made and the characteristics of the context. | 6.1 Presents information about the work done through digital media that are available. |

| **METHODOLOGY: Proposed Methodologies for community work**.  ● Small group work  ● Online interviews  ● Online focus groups  ● Problem tree  ● Brainstorming  ● Infographics  ● Synchronous educational sessions  ● Videos  ● Information through audios |
| --- |

| \| **COURSE EVALUATION**  Students should be familiar with the Evaluation Rubrics from the beginning of the course and their final evaluation prior to the submission of the Course Outline.  **Teamwork**  Rubric Teamwork **35%**  **Personal work**  Rubric "Audio about work experience in the community and with their team" **15%**  **Community intervention**  Rubric for educational intervention in health **50%** \|  \| \| --- \| --- \| |
| --- | --- | --- |

| **BIBLIOGRAPHY .**  **Basic Bibliography:**   1. Vásquez. M, Acevedo. J, Olave. V, Aguilera. H, Sandoval . D, Ahlers. I, Jorquera. M, Muñoz.C. Guía para el desarrollo de intervenciones educativas en salud en la comunidad. APSF. Facultad de Medicina. Universidad de Chile. 2016. 2. López. L, Covarrubias. S. MINSAL. Manual de Trabajo Comunitario. Postas de Salud Rural. 2010.   **Supplementary Bibliography**   1. Oliver Gröne, MilaGarcia-Barbero. Integrated Care. *International Journal of Integrated Care*. Vol. 1. 1 – 10. 2001 2. WHO. The solidFacts. Social Determinants of Health. 2003 3. Ivy Lynn Bourgeault; GillianMulvale. Collaborative Healthcare Teams in Canada and the USA: Con- fronting the structural embeddedness of medical dominance. *Health SociologyReview;* 15, 5*.* 2006 4. Abu-Rish E., Kim S., Choe L., Varpio L., Malik E., White A.A., Zierler B. (2012). Current Trends in interprofessional education of health sciences students: a literature review*. Journal of Interprofessional Care*, *26* (6), 444-51. 5. Hogden A., Greenfield D.,Nugus P., Kiernan MC. (2012). Engaging in patient decision-making in multidisciplinary care for amyotrophic lateral sclerosis: the views of health professionals. *PatientPreference and Adherence*, *6*, 691-701. 6. IOM [Institute of Medicine], (2015). *Measuring the impact of interprofessional education collaborative practice and patient outcomes*. Washington, DC.: The NationalAcademiesPress. 7. Reeves S., Perrier L., Goldman J., Freeth D., &Zwarenstein M. (2013). Interprofessional education: Effects On Professional practice and healthcare outcomes (update) (Review). *The Cochrane Library*, Issue 3. DOI: 10.1002/14651858.CD002213.pub3. 8. Rodríguez D., Berenguera A., Pujol-Ribera E., Capella J., Peray J.L., & Roma J. (2013). *Identificación de las competencias actuales y futuras de los profesionales de la salud pública*. Gaceta Sanitaria, *27* (5), 388-97. 9. Joseph, S., Diack, L., Garton, F., and Haxton, J. (2012). *Interprofessional education in practice*. ClinTeach 9, 27-31 10. Thistletwaite, J. (2012) *Interprofessional education: a review of context, learning and the research agenda*. Medical Education 46: 58–70. 11. [Olson, R](http://www.ncbi.nlm.nih.gov/pubmed/?term=Olson%20R%5BAuthor%5D&cauthor=true&cauthor_uid=24528458). [Bialocerkowski, A](http://www.ncbi.nlm.nih.gov/pubmed/?term=Bialocerkowski%20A%5BAuthor%5D&cauthor=true&cauthor_uid=24528458). (2014) *Interprofessional education in allied health: a systematic*review.[Med Educ.](http://www.ncbi.nlm.nih.gov/pubmed/24528458)48(3):236-46. doi: 10.1111/medu.1229 12. [Reeves, S](http://www.ncbi.nlm.nih.gov/pubmed/?term=Reeves%20S%5BAuthor%5D&cauthor=true&cauthor_uid=23543515)., [Perrier, L](http://www.ncbi.nlm.nih.gov/pubmed/?term=Perrier%20L%5BAuthor%5D&cauthor=true&cauthor_uid=23543515)., [Goldman, J](http://www.ncbi.nlm.nih.gov/pubmed/?term=Goldman%20J%5BAuthor%5D&cauthor=true&cauthor_uid=23543515)., [Freeth, D](http://www.ncbi.nlm.nih.gov/pubmed/?term=Freeth%20D%5BAuthor%5D&cauthor=true&cauthor_uid=23543515)., [Zwarenstein, M](http://www.ncbi.nlm.nih.gov/pubmed/?term=Zwarenstein%20M%5BAuthor%5D&cauthor=true&cauthor_uid=23543515). (2013) *Interprofessional education: effects on professional practice and healthcare outcomes*. [Cochrane DatabaseSyst Rev.](http://www.ncbi.nlm.nih.gov/pubmed/23543515)28;(3): CD002213. doi: 10.1002/14651858.CD002213.pub3. 13. Interprofessional Education CollaborativeExpert Panel. (2011). Core Competencies for interprofessional collaborative practice: Report of an expert panel. Washington, D.C.: Interprofessional Education Collaborative.Downloaded 21 december 2013 from   <https://www.aamc.org/download/186750/data/core_competencies.pdf>   1. OPS. Salud en Chile 2010. Panorama de la situación de salud y del sistema de salud en Chile. 2011 2. Julio Frenk*, Lincoln Chen*, Zulfiqar A Bhutta, Jordan Cohen, Nigel Crisp, Timothy Evans, Harvey Fineberg, Patricia Garcia, Yang Ke, Patrick Kelly,Barry Kistnasamy, Afaf Meleis, David Naylor, Ariel Pablos-Mendez, Srinath Reddy, Susan Scrimshaw, Jaime Sepulveda, David Serwadda,Huda Zurayk. Health professionals for a new century: transforming education to strengthen health systems in an interdependent world. *The lancet*. Vol 376:1923 – 1958. 2010 3. *Schejter, Virginia1; Selvatici, Laura2; Cegatti, Julia3; De Raco, Paula Paulette4; Ugo, Florencia5; Je- siotr, Malena6.* IMAGINARIZAR EL TRABAJO INTERDISCIPLINARIO EN SALUD. Vol XIII: 195 – 200. 2005 4. OMS.Educación para la salud : Manual sobre educación sanitaria en atención primaria de salud .OMS 5. MINSAL. Manual de apoyo a la incorporación de agentes comunitarios en salud en los equipos de los CECOSF. 2016 6. MINSAL. PARTICIPACIÓN CIUDADANA EN ATENCIÓN PRIMARIA Un aporte al desarrollo del Modelo de Atención Integral de Salud familiar y comunitario y la satisfacción usuaria.2016 |
| --- |

| **PASSING REQUIREMENTS**  Article 24: The academic performance of the students will be graded on a scale of grades from 1.0 to 7. The minimum passing grade for each of the curricular activities for all purposes will be 4.0, with approximation.  Partial grades, final activity grades and the final activity grade will be given to the nearest hundredth of a point. The final grade of the curricular activity shall be given to one decimal place for passing grades, in which case 0.05 or higher shall be approximated to the upper digit and less than 0.05 to the lower digit.  Article 26: The grade of the curricular activity will be made on the basis of the achievements evidenced by the student in the competencies established in them. The final grade of the various courses and curricular activities will be obtained from the weighting of the grades of each learning unit and the final activity of the course if any.  The minimum passing grade is 4.0 and each course syllabus must specify the requirements and conditions of approval prior acceptance by the School Board.  Given the particular situation of Pandemic, the decision was made for this year 2020, applicable to courses with final exam.  The exam does not have a failing grade, that is to say, if the student has a sufficient grade to take the exam and obtains a grade lower than 4 on it, but it is weighted above 4.0, he/she approves the course.  General Regulations of the training plans leading to the degrees and professional titles granted by the Faculty of Medicine, D.U. Nº003625 of January 27, 2009 |
| --- |

| **POLICY OF SOCIAL CO-RESPONSIBILITY IN THE RECONCILIATION OF FAMILY RESPONSIBILITIES AND UNIVERSITY ACTIVITIES.**  With the purpose of fulfilling the objectives of: To tend to overcome the cultural and institutional barriers that prevent the full deployment, under equal conditions, of women and men in the University and the country; To guarantee equal opportunities for the equitable participation of men and women in different areas of university activities; Develop measures and actions that favor social co-responsibility in the care of children and allow the reconciliation of work, student and family life; and, Develop a pertinent regulatory framework through the study and analysis of current university regulations and their eventual modification, as well as the creation of new regulations and general norms related to the University's policies and development plans; five complementary lines of action are contemplated:  Line of Action N°1: provide care and initial education services to children of students, academics and collaboration personnel, thus facilitating the exercise of their work or study roles and functions, through the installation of crèches and public kindergartens in the various university campuses.  Line of Action No. 2: favor the conciliation between the performance of student and family responsibilities, by establishing in the university regulations criteria that allow students to obtain the necessary assistance from the academic units within the framework of social co-responsibility in the care of children.  Line of Action N°3: guarantee gender equity in the evaluation and academic qualification processes, through the adaptation of the respective university regulations, in order to allow equal opportunities between academics in the different instances, considering the effects of maternity and family responsibilities in the performance and productivity, both professional and academic, as appropriate. |
| --- |

| **UNIVERSITY POLICY ON INCLUSION AND FUNCTIONAL DIVERSITY**  Area of Action C: Universal Accessibility  The University must safeguard universal accessibility in all events and physical and virtual spaces, so that all members of the university community can access and actively participate in all the opportunities that our institution offers.  It should be considered that part of universal accessibility has to do with the proper installation of signage and the provision of accessible toilets in all spaces where people with disabilities must stay or transit.  In the main official activities of the University, there must be a sign language interpreter that allows free participation without discrimination to those students and members of the university community who are hearing impaired.  a) INFRASTRUCTURE: In the new infrastructure as well as in the already built and heritage infrastructure, accessibility must be safeguarded for all people who need or wish to participate in the activities that take place there, ensuring that none of them arbitrarily prevent the access and participation of people.  b) INFORMATION: The information emanating from the University must be accessible to all people, contemplating the possibility of specific readings for people with hearing or visual disabilities. Similarly, communication and training platforms must be designed or adapted under the perspective of universal design and accessibility.  c) TRAINING: Although initially in a transformation process for the inclusion of people with disabilities, adaptations and adaptations (structural, virtual, educational) are necessary, training in the perspective of Universal Design, both infrastructure and communication platforms and learning strategies should be aimed at. |
| --- |

**ATTENDANCE REGULATIONS**

Compulsory activities

Duly justified absences to these activities must be made up according to the availability of time, teachers and clinical field. If, due to their nature or amount, they cannot be made up, the student must take the course in its entirety in the next academic period, as Pending or Failed, as appropriate.

a) The student who exceeds the maximum number of absences allowed, will appear as "Pending" in the Final Grade of the course, provided that in the opinion of the PEC, or the Level Council or the School Board, the absences with due cause (e.g., verifiable medical certificate, SEMDA report, social or family reasons accredited by the Student Welfare Service).

b) The student who exceeds the maximum number of absences allowed, and did not provide reasonable and sufficient evidence to justify the number of absences, will appear as "Failed" in the final grade of the course with a final grade of 3.4.

Evaluations

Non-attendance to an evaluation must be communicated by the most expeditious means (telephone - electronic) within 24 hours after the date of the scheduled activity.

The justification for non-attendance must be presented to the School Secretary's Office within 5 working days from the date of non-attendance, certified by the authorized Services of the Faculty: Student Medical and Dental Service, Student Welfare Service and School Direction.

If the justification is made within the stipulated deadlines and your PEC accepts the justification, the activity must be recovered preferably orally in front of the committee and cumulative.

If this justification is not made within the stipulated deadlines, the student must be graded with the minimum grade (1.0) in that evaluation activity.

General Regulations of Studies of the School of Medicine, D.E. No. 0010109 of August 27, 1997.

Resolution No. 1466 "Operative norm on non-attendance to mandatory curricular activities for undergraduate students of the School of Medicine. October 16, 2008.

| **MIIM 2 ON LINE 2020 CALENDAR OF ACTIVITIES** | | | | |
| --- | --- | --- | --- | --- |
| 1 | November 10th | 15:00 -18:00 hrs | Work with Teacher and social leader in the community.  Introduction MIIM 2 course syllabus and evaluation system. | MIIM2 Teams |
| 2 | November 17th | 15:00 -18:00 hrs | Work with Teacher and Social Leader in the community.  Work analyzing information about the assigned community in the field. | MIIM2 Teams |
| 3 | November 24th | 15:00 -18:00 hrs | Working with Teacher and Social Leader in the community | MIIM2 Teams |
| 4 | December 1st | 15:00 -18:00 hrs | Working with Teacher and Social Leader in the community | MIIM2 Teams |
| 5 | December 15th | 15:00 -18:00 hrs | Working with Teacher and Social Leader in the community | MIIM2 Teams |
| 6 | December 22nd | 15:00 -18:00 hrs | Working with Teacher and Social Leader in the community | MIIM2 Teams |
| 7 | January 5th,2021 | 15:00 -18:00 hrs | Working with Teacher and Social Leader in the community  Final assessment in each team | MIIM2 Teams |
| 8 | January 12th, 2021 | 15:00 -18:00 hrs | Working with Teacher and Social Leader in the community  Final assessment in each team | MIIM2 Teams |
