# Supplementary figures and images for: Online interprofessional education materials through a community learning program during the COVID 19 pandemic in Chile
Source: J Educ Eval Health Prof. 2022 Mar 24;19:6. doi: 10.3352/jeehp.2022.19.6 (PMC9086809; doi:10.3352/jeehp.2022.19.6)

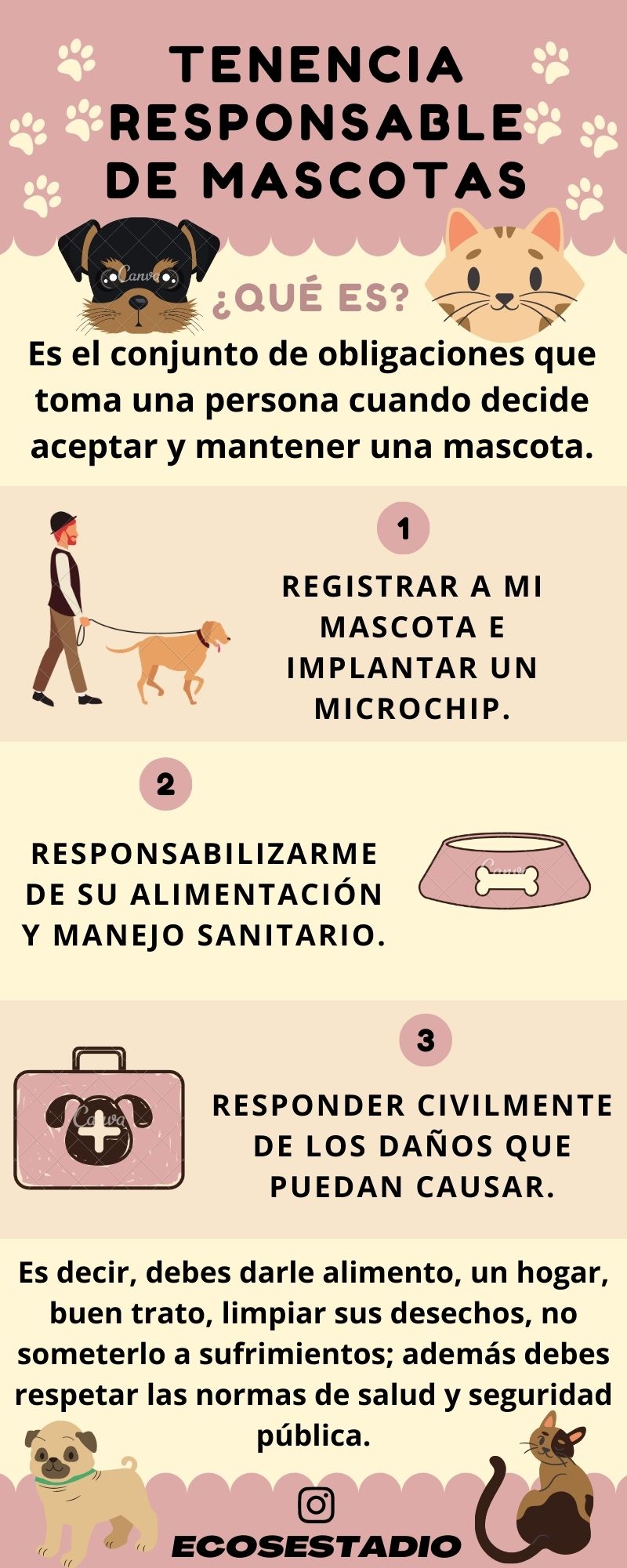

Supplement: Supplementary file 2 — Supplement 2. Example of Interprofessional education infographics “Infografía Tenencia Responsable de mascotas” used by students to educate community groups (in Spanish)The infographics developed by the students included different educational topics requested by the community, such as responsible pet ownership [file jeehp-19-06-suppl2.jpg]
